# Identification of genomic regions regulating sex determination in Atlantic salmon using high density SNP data

**DOI:** 10.1186/s12864-019-6104-4

**Published:** 2019-10-22

**Authors:** María Gabián, Paloma Morán, Ana I. Fernández, Beatriz Villanueva, Amel Chtioui, Matthew P. Kent, Lara Covelo-Soto, Almudena Fernández, María Saura

**Affiliations:** 1 Departamento de Bioquímica, Genética e Inmunología, Facultad de Biología, Universidad de Vigo, Vigo, 36310 Spain; 2 Departamento de Mejora Genética Animal, INIA, Carretera de la Coruña km 7,5, 28040 Madrid, Spain; 3 Center for Integrative Genetics (CIGENE), Department of Animal and Aquacultural Sciences, Faculty of Bioscience, Norwegian University of Life Sciences (NMBU), 1430 Ås, Norway

**Keywords:** Atlantic salmon, GWAS, High-dense SNP chip, Regional heritability analysis, Sex determination

## Abstract

**Background:**

A complete understanding of the genetic basis for sexual determination and differentiation is necessary in order to implement efficient breeding schemes at early stages of development. Atlantic salmon belongs to the family Salmonidae of fishes and represents a species of great commercial value. Although the species is assumed to be male heterogametic with XY sex determination, the precise genetic basis of sexual development remains unclear. The complexity is likely associated to the relatively recent salmonid specific whole genome duplication that may be responsible for certain genome instability. This instability together with the capacity of the sex-determining gene to move across the genome as reported by previous studies, may explain that sexual development genes are not circumscribed to the same chromosomes in all members of the species. In this study, we have used a 220 K SNP panel developed for Atlantic salmon to identify the chromosomes explaining the highest proportion of the genetic variance for sex as well as candidate regions and genes associated to sexual development in this species.

**Results:**

Results from regional heritability analysis showed that the chromosomes explaining the highest proportion of variance in these populations were Ssa02 (heritability = 0.42, SE = 0.12) and Ssa21 (heritability = 0.26, SE = 0.11). After pruning by linkage disequilibrium, genome-wide association analyses revealed 114 SNPs that were significantly associated with sex, being Ssa02 the chromosome containing a greatest number of regions. Close examination of the candidate regions evidenced important genes related to sex in other species of Class Actinopterygii, including *SDY*, genes from family *SOX, RSPO1, ESR1, U2AF2A, LMO7, GNRH-R, DND* and *FIGLA*.

**Conclusions:**

The combined results from regional heritability analysis and genome-wide association have provided new advances in the knowledge of the genetic regulation of sex determination in Atlantic salmon, supporting that Ssa02 is the candidate chromosome for sex in this species and suggesting an alternative population lineage in Spanish wild populations according to the results from Ssa21.

## Background

Sexual development is a multistep process involving sex determination (SD), initiation, gonadal differentiation and maintenance. This process comprises a great diversity of strategies that can be controlled by a variety of genetic and/or environmental mechanisms [1–3].

Characterising the genetic basis of SD in fish is fundamental for broodstock management in breeding programmes, which require controlling the entire life cycle of the animal, particularly reproduction. Gaining control over SD enables to develop appropriate breeding schemes in early stages of development when sex external secondary characteristics are not yet differentiated [4, 5]. In this regard, sex control is needed to prevent precocious maturation and reduce the impact of phenotypic sex on product quality; to produce monosex populations when there are differences in growth rate between sexes; to favor the stability of mating systems or to protect wild populations through supportive breeding practices, as having control on the sex ratio if fundamental to maintain the effective population size and therefore to avoid inbreeding depression [6].

The family Salmonidae (Salmonids) comprises fish species with a great economic and societal importance. Within them, Atlantic salmon (*Salmo salar*) represents one of the most important farmed fish species in the world, with a global annual production achieving 2.5 million tonnes that entails an economic value of $15.4 billion (USD) [7].

Based on available evidence, it is assumed that salmonids are male heterogametic and that sex determination is genetically controlled by the master-sex *SDY* gene (sexually dimorphic on the Y-chromosome), a gene from the interferon-response factor transcription family [8, 9], which is involved in the immune response of vertebrates. In many non-salmonid species, master-sex determining genes are located on differentiated sex chromosomes that have undergone reduced recombination around the areas of the determining gene, leading to heteromorphic sex chromosomes [10, 11]. However, in Atlantic salmon, as in other salmonid species, sex chromosomes are not morphologically distinguishable [12]. Despite the fact that *SDY* is associated to maleness in most salmonids [9], its location is not syntenically conserved among species. Although previous literature supports that in Atlantic salmon, *SDY* maps to chromosome Ssa02 [13, 14], in some individuals of the species it has been found mapping to different chromosomes [15–18]. These findings have been associated to a transposition ability of *SDY* between chromosomes [14, 19], but the underlying mechanisms for this mobility are still unclear. A recent study by Kijas et al. [17] found evidence of one single ancestral location for Atlantic salmon *SDY,* thus discarding the hypothesis of multiple genomic locations predating Atlantic salmon speciation. Other studies have suggested that salmonids’ genome is at an early stage of sex chromosome evolution, given its residually tetraploid state resulting from the salmonids extra whole genome duplication (WGD) [12, 14]. The phenomenon, residual tetrasomy [20, 21], explains how some telomeric regions continue recombining between homeologous chromosomes while others have rediploidized [20–22], which may facilitate the transposition of genes across the genome and delaying Y degeneration [9, 16, 18]. This delay in Y degeneration has proposed to be mediated by sex reversal events in fish [23], as a consequence of the formidable plasticity in SD mechanisms in this group. In this way, sex reversal might play an important role in the evolution of SD, facilitating the purge of deleterious mutations on the heterogametic sex chromosome through recombination. This has been proposed as a “fountain of youth” [24] that may explain the high incidence of homomorphic sex chromosomes in fish and amphibians.

The advent of next-generation sequencing technologies has facilitated the development of a high quality reference genome [25] and multiple high-density single nucleotide polymorphism (SNP) arrays [26–28] for Atlantic salmon. The advance of molecular tools has also entailed the development of new statistical approaches that open new opportunities for the investigation of complex traits in this species. In this sense, the regional heritability analysis (RHA) approach, recently proposed by Nagamine et al. [29] represents an appropriate methodology to obtain a first approximation of the role of the different chromosomes of Atlantic salmon in sex determination.

In this study, we have used a 220 K high-density SNP panel [27] to investigate the genetic regulation of sex determination in wild Spanish populations of Atlantic salmon, which inhabit the South distribution limit of the species in Europe. The combination of RHA and genome-wide association studies (GWAS) allowed us to determine which chromosomes explain the highest proportion of the genetic variance for sex as well as identify candidate regions and genes associated to sexual development in this species.

## Results

### Regional heritability analysis

**Fig. 1:**
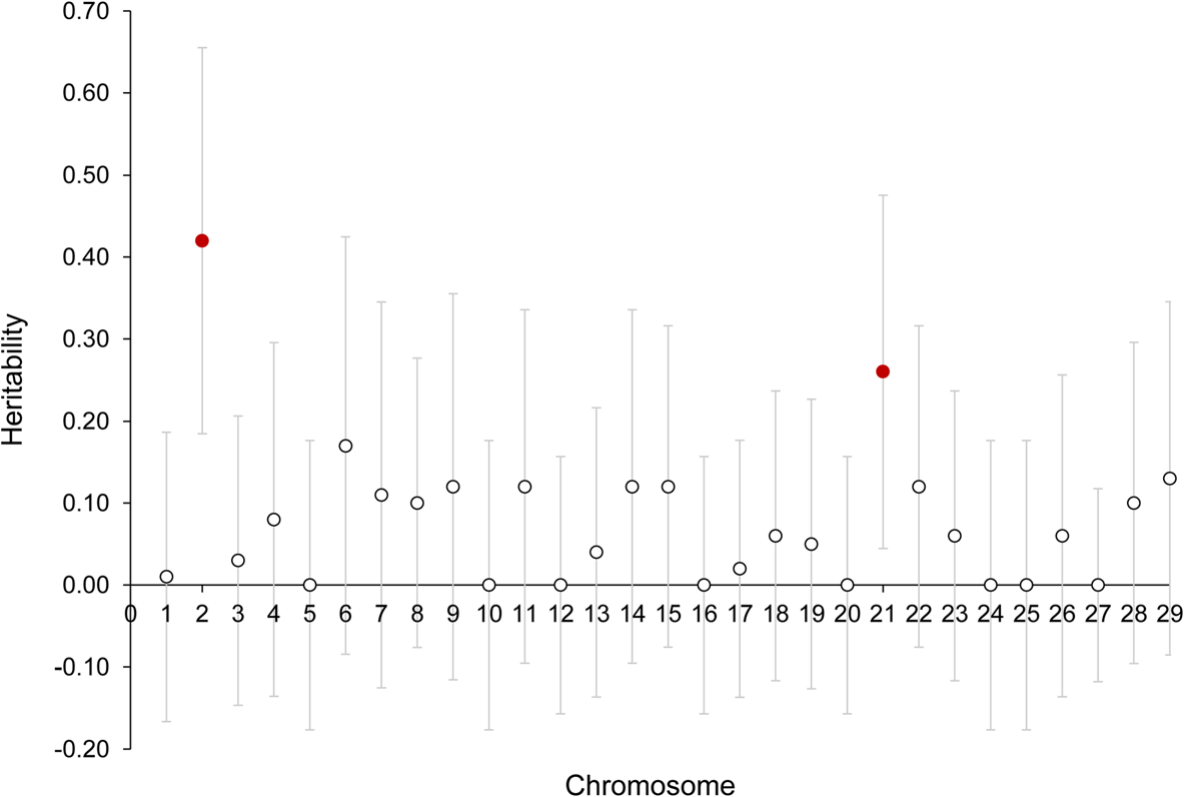
Heritability estimates for each chromosome. Estimates of heritability (*h*^2^) for sex and corresponding confidence intervals at 95% level (in red those significantly different from zero)

Genomic heritability for sex in the group of individuals analysed was significant and high (*h*^*2*^ = 0.70, SE = 0.26). Estimates of chromosomal heritability ranged from 0.00 to 0.42 and are summarised in Table 1. Only estimates from chromosomes Ssa02 (*h*^*2*^ = 0.42, SE = 0.12) and Ssa21 (*h*^*2*^ = 0.26, SE = 0.11) were significant at the chromosomal (suggestive) level, as revealed by Likelihood Ratio Tests (LRT) (Table 1) and 95% confidence intervals (Fig. 1). After applying the strict Bonferroni correction for multiple test (5% level), the estimate of heritability for Ssa02 was still significant. This chromosome explained 60% of the total additive genetic variance for sex in these populations.

**Table 1:** Whole-genome (genomic) and chromosomal heritability estimates for sex

Chromosome	*h* ^*2*^	SE	LRT	p-val
1	0.01	0.09	―0.09	―
2	0.42	0.12	22.56	1.02e-6
3	0.03	0.09	―0.24	―
4	0.08	0.11	0.01	0.4586
5	0.00	0.09	―0.73	―
6	0.17	0.13	0.56	0.2275
7	0.11	0.12	0.19	0.3322
8	0.10	0.09	0.00	―
9	0.12	0.12	0.21	0.3224
10	0.00	0.09	―2.15	―
11	0.12	0.11	0.22	0.3209
12	0.00	0.08	―0.91	―
13	0.04	0.09	―0.07	―
14	0.12	0.11	0.23	0.3166
15	0.12	0.10	0.93	0.1681
16	0.00	0.08	0.00	―
17	0.02	0.08	0.03	0.4292
18	0.06	0.09	0.01	0.4574
19	0.05	0.09	0.00	0.4865
20	0.00	0.08	―0.82	―
21	0.26	0.11	6.27	0.0062
22	0.12	0.10	0.38	0.2687
23	0.06	0.09	0.07	0.3934
24	0.00	0.09	0.12	0.3665
25	0.00	0.09	―0.14	―
26	0.06	0.10	0.00	―
27	0.00	0.06	―3.36	―
28	0.10	0.10	0.55	0.2302
29	0.13	0.11	1.23	0.1338
Genomic	0.70	0.26		

Estimates of heritability (*h*^2^) for sex and corresponding standard error (SE) for each of the chromosomes and for the whole genome. Likelihood ratio test (LRT) statistic derived from the comparison between the reduced and the full models, with corresponding p-values, are also indicated

### Genome-wide association analysis

**Fig. 2:**
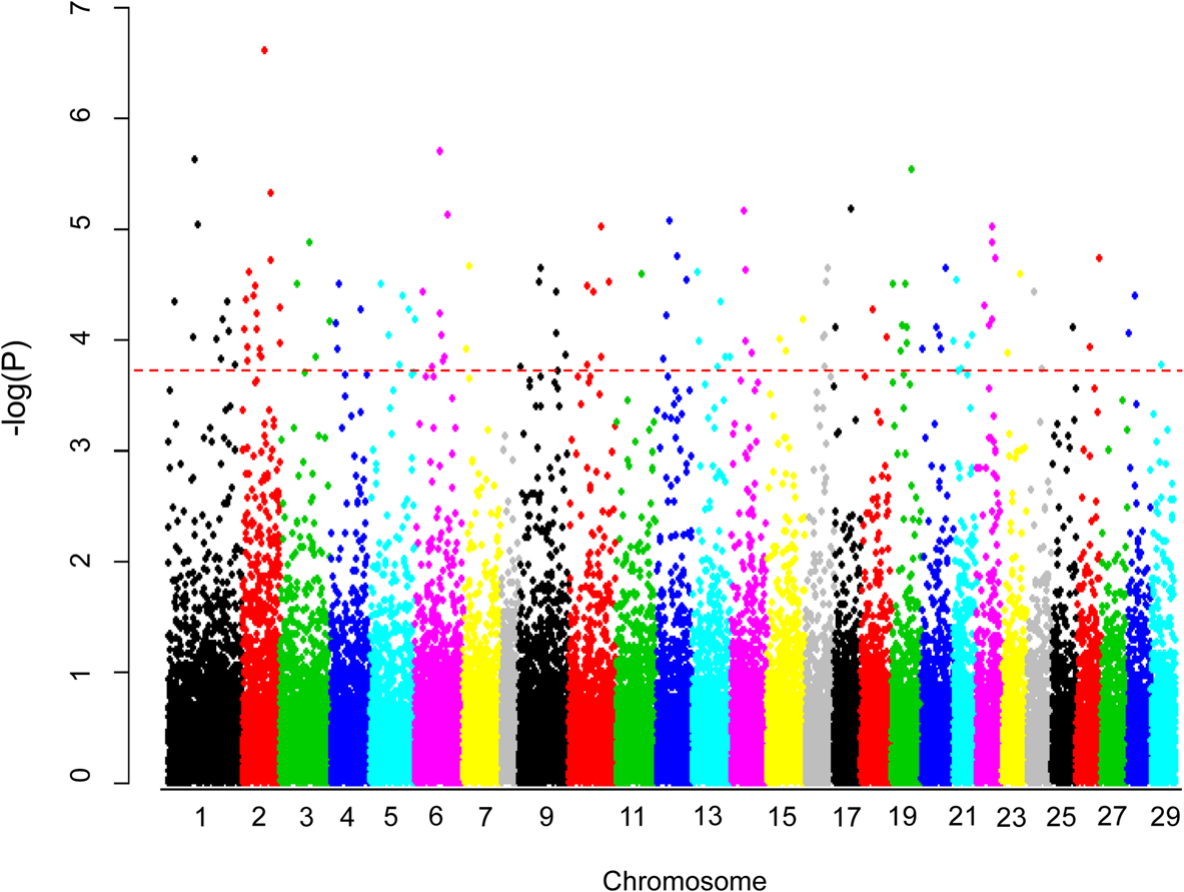
Manhattan plot resulting from the GWAS for sex at 5% false discovery rate (FDR) multitest correction threshold. Log transformed *p*-values are represented in the Y-axis, against the position of the SNP in the chromosome (X-axis). Red discontinuous line represents FDR multitest correction threshold at 5% level

Results derived from GWAS after pruning the SNPs for linkage disequilibrium (LD) and after applying false discovery rate (FDR) multitest correction at 5% level (Log_10_ (P) > 3.8), revealed 114 SNPs significantly associated with sex (Fig. 2, Table 2). Ssa02 was the chromosome where the highest number of significant SNPs were mapped (15%). Allele frequencies for the significant SNPs were in general intermediate, as expected given the balanced numbers of males and females in the data set analysed, with SNP effects ranging between ―0.29 and 0.33 (Additional file 1A). The distribution of allele frequencies for significant SNPs revealed a generalized differential trend for males and females (Additional file 1B). Further information about SNP location on chromosomes and corresponding regions is available in Additional file 1.

**Table 2:** Information on the number of significant SNPs identified in the GWAS for each chromosome

Chromosome	Length	*N SNPs*	
		Pruned (Total)	Significant
1	159.0	2700 (12,768)	9
2	72.9	1357 (4689)	17
3	92.5	1846 (7940)	4
4	82.4	1435 (6302)	4
5	80.5	1619 (6455)	5
6	87.0	1756 (6966)	7
7	58.8	1374 (4894)	2
8	26.4	646 (1725)	0
9	141.7	1821 (9762)	5
10	116.1	1723 (8180)	5
11	93.9	1473 (6526)	1
12	91.9	1275 (5841)	5
13	107.8	1435 (7335)	5
14	93.9	1297 (6852)	4
15	104.0	1396 (7007)	3
16	87.8	1035 (5558)	4
17	57.7	982 (3736)	2
18	70.7	1128 (5401)	2
19	83.0	1086 (5369)	7
20	86.8	1167 (5762)	5
21	58.0	824 (4010)	4
22	63.4	941 (4832)	6
23	49.9	922 (4108)	2
24	48.7	887 (4415)	1
25	51.5	892 (4201)	1
26	47.9	906 (3605)	2
27	43.9	962 (4001)	0
28	39.6	847 (3233)	2
29	42.5	893 (3410)	0
Genomic		36,625 (164,883)	114

For each chromosome, a summary of the length in Mb, number of SNPs analysed after (Pruned) and before applying the pruning filter for linkage disequilibrium (Total, in brackets), and the number of significant SNPs in the GWAS are indicated

### Functional analysis

We first used the information available in the SalmoBase database for Atlantic salmon to explore the gene content of the 17 candidate regions identified on Ssa02 (10 regions, including five overlapping regions) and Ssa21 (four regions), the chromosomes showing significant (and highest) estimates of heritability. SalmoBase contains genome annotation information obtained from RNAseq data, including 48,775 protein coding genes identified to date [25]. Our search revealed 543 and 62 genes contained within candidate regions for chromosomes Ssa02 and Ssa21, respectively (Table 3, Additional file 2). However, the still early stages of annotation of the Atlantic salmon genome, hampered gene enrichment analysis for identifying metabolic routes associated to sex determination.

**Table 3:** Information of the candidate regions identified in the GWA analysis in chromosomes Ssa02 and Ssa21 and corresponding number of genes in *Salmo salar* annotation

Chrom	Region	Start	End	N genes
2	10	5,577,222	6,577,222	42
2	11	10,142,805	12,197,890	102
2	12	13,985,694	14,985,694	49
2	13	22,816,807	24,177,902	36
2	14	26,416,426	27,936,237	73
2	15	30,702,935	33,299,026	47
2	16	43,765,979	44,765,979	36
2	17	51,194,734	52,194,734	39
2	18	52,856,992	53,856,992	47
2	19	70,787,705	72,294,817	72
21	90	448,663	1,448,663	12
21	91	5,232,214	6,232,214	19
21	92	25,444,577	26,444,577	17
21	93	46,118,091	47,118,091	14

Region code and corresponding start and end positions (in bp) of the candidate regions identified in chromosomes Ssa02 and Ssa21 in the GWAS. The number of genes found in the *Salmo salar* annotation from Salmobase is also indicated

For this reason, an alternative strategy was performed, consisting on BLAST search of sex related genes previously identified in Atlantic salmon [30] and in other species of the Class Actinopterygii. From the 74 genes initially explored, 56 genes from 12 species aligned against the *Salmo salar* genome with a query cover > 50% (Additional file 3). Those with lower query cover were genes from non-salmonid species. Imperfect query cover was also allowed for sex-related genes from Atlantic salmon, in order to detect mapping of these genes to alternative regions due to replication or recombination in our population. Thirteen of these 74 genes were located within or close to candidate regions previously identified in the GWAS (Table 4), including important genes related to sex in other species, such as: *SDY*, genes from the *SOX* family (*SOX1, SOX1b, SOX8, SOX9, SOX21, SOX21a*), *RSPO1*, *ESR1*, *U2AF2A*, *LMO7, GNRH-R*, *DND* and *FIGLA* (Table 5).

**Table 4:** Summary of sex related genes from other species of Class Actynopterigii located in candidate regions in the *Salmo salar* genome

Chrom	N genes	Region	Start (bp)	End (bp)	Gene	Organism
2	20	10	4,378,828	4,378,974	*RSPO1*	*Oryzias latipes*
		10	4,384,694	4,384,886	*ESR1*	*Danio rerio*
		10^1^	6,070,144	6,070,307	*SOX8*	*Esox lucius*
		11^1^	11,563,571	11,564,290	*SDY*	*Oncorhynchus mykiss*
3	2	–				
4	1	–				
5	6	–				
6	7	–				
7	1	–				
9	2	–				
10	7	–				
12	1	–				
13	2	–				
14	2	66^1^	4,239,034	4,365,414	*U2AF2A*	*Danio rerio*
15	8	–				
16	1	–				
17	2	–				
18	3	–				
19	4	83	3,430,459	3,431,574	*SOX9*	*Cynoglossus semilaevis*
20	4	87	3,469,065	3,469,779	*SDY*	*Oncorhynchus mykiss*
21	10	90^1^	453,741	454,610	*LMO7*	*Salvelinus alpinus*
		90^1^	1,371,035	1,371,951	*SOX21*	*Poecilia reticulata*
		90^1^	1,371,136	1,371,950	*SOX21A*	*Danio rerio*
		90^1^	1,371,677	1,371,943	*SOX1B*	*Danio rerio*
		90	1,507,905	1,508,982	*SOX1*	*Poecilia reticulata*
22	2	**–**				
23	7	98	9,548,635	9,549,406	*GNRH-R*	*Oncorhynchus mykiss*
24	3	–				
25	3	–				
26	1	–				
27	3	–				
28	7	–				
29	1	–				

Number (N) of sex related genes mapped in this study for each chromosome (Chrom). For those genes located within ^1^ candidate regions predefined (< 0.5 Mb from the significant SNP) or in the vicinity of candidate regions (< 2.5 Mb from the significant SNP), the specific location of the gene in the *S. salar* genome and the organism where the gene was identified in NCBI are also detailed

**Table 5:** Sex-related genes previously described in Actinopterygii that matched with GWAS candidate regions identified in this study

Genes	Location	Functional group	Functional role in sex	Reference
*SDY*	Ssa02, 19, 20	Interferon regulatory factor	Up-regulation of inhibitors of *CYP19A* (cytochrome P450 aromatase), preventing the accumulation of estrogens required for female development.	[8, 9, 16]
*SOX* family	Ssa01–3, 5–7, 9–10, 12–17, 19, 21–23, 25–29	Transcription factors	Testis development and male fertility in mammals. Sertoli cell differentiation and seminiferous tubule formation.	[31, 32]
*RSPO1*	Ssa02, 5	Activator protein	Activator of ovarian determination and differentiation through the *WNT/β-catenin* signaling pathway.	[33, 34]
*LMO7*	Ssa21, 25	Protein-protein interactions	Regulation of transcription of the emerin gene, an inhibitor of the *WNT/β-catenin* signaling pathway through beta-catenin.	[35, 36]
*ESR1*	Ssa02, 6, 15	Ligand-activated transcription factor	Estrogen nuclear receptor involved in female development in vertebrates. Maintenance of development and maintenance of female status.	[37, 38]
*U2AF2A*	Ssa02, 14, 15, 27	Binding factor	Steroid receptor. Regulation of transcription mediated by steroid hormone receptors and alternative sex-specific splicing in vertebrates. Female sex differentiation.	[39]
*GNRH-R*	Ssa02, 5, 23	Binding factor	Receptor of gonadotropin-releasing hormone. Regulation of female reproduction in vertebrates.	[40, 41]
*DND*	Ssa17, 21	Binding factor	Germ-cell-marker gene in many vertebrates. Involved in primordial germ cell migration and survival in teleosts.	[42]
*FIGLA*	Ssa02, 20, 21	Transcription factor	Germ-cell-specific transcription factor associated with ovary development and differentiation in vertebrates.	[43]

For each gene, the chromosomal location in Atlantic salmon candidate regions identified in this study, the functional group to which the gene belongs to, its functional role in sex and corresponding references, are provided

## Discussion

In this study, we used two complementary approaches to improve our understanding of the genetic mechanisms responsible for sex determination in Atlantic salmon. The information obtained from a high-density SNP array was used to perform a RHA that allowed to obtain, for the first time in this species, chromosomal estimates of heritability for sex. Furthermore, this information was used to identify putative regions responsible for sex determination using GWAS. By combining both approaches, our results support that Ssa02 is the predominant sex-determining chromosome in native Spanish populations, in agreement with much of the existing literature in other Atlantic salmon populations.

Previous studies seeking to identify sex related QTLs in Atlantic salmon, based their work on linkage map regression methods using a lower number of markers and cytogenetic techniques [13, 44, 45]. These studies suggested that Ssa02 contained the *SEX* locus, a finding that was later confirmed by the discovery of the *SDY* master-sex determining gene in rainbow trout [8, 9]. Despite today is generally accepted that *SDY* is responsible of maleness in most salmonids, some exceptions have been described. Hence, analysing families of a commercial Tasmanian Atlantic salmon population of North American origin, Eisbrenner et al. [15] mapped the *SEX* loci on three different chromosomal locations (Ssa02, Ssa03 and Ssa06). A recent study in the same population by Kijas et al. [17] confirmed, through whole-genome sequencing, that Atlantic salmon males carry a single copy of the male-sex determining region containing *SDY*. Although it was identified on chromosome Ssa02 in most of individuals, some animals presented this region on Ssa03 and Ssa06, supporting the results by Eisbrenner et al. [15]. Also through genome-wide association, the authors identified candidate regions for four additional chromosomes.

Although our GWAS pattern was more sparse than that from Kijas et al. [17], we identified the same candidate regions in five chromosomes, including Ssa02 (regions 13–17 in Additional file 1), Ssa03 (22–23), Ssa05 (30–32), Ssa06 (37–39) and Ssa12 (53). The different association patterns observed in both studies can however be explained by different technical and biological reasons. First, the 220 K SNP used in our study was designed with samples from Norwegian origin, thus SNPs segregating in both populations are expected to be different. Although the SNP content from the 50 K SNP chip used by Kijas et al. [17] largely derived from the 220 K SNP chip, it was designed for identifying segregating polymorphic loci in the Tasmanian population, thus maximising the amount of information. Second, we pruned our data set to avoid overweighting the contribution of groups of correlated SNPs due to linkage disequilibrium that might contribute to lower accuracy [46]. For that, we imposed a strong filter based on half the maximum value for *r*^*2*^ observed in this population (Additional file 4), which can affect the pattern observed. Indeed, linkage disequilibrium patterns might be considerably different in both populations, since they have different geographic origin (South European vs North American) and management (wild vs farmed), and therefore are subjected to completely different selection pressures. Our wild populations inhabit the South distribution limit of the species in Europe, where strong local adaptations are expected. Conversely, the samples from Kijas et al. [17] come from a Tasmanian breeding programme of Nova Scotia origin, that has been maintained isolated for generations, where the impact of artificial selection and genetic drift is expected to have affected the distribution of the genetic variability across the genome in a different manner than in wild populations. In addition, due to its strong homing behaviour, the Atlantic salmon is naturally substructured into genetically differentiated and reproductively isolated populations [47]. Previous studies investigating the population structure of this species have found evidence of substantial genetic differentiation between North American and European populations (with 22% of the variation attributable to continents), with a clear pattern of isolation by distance [48]. Within continents, salmon European populations are more divergent than North American ones and are differentiated in Eastern Atlantic and Baltic clusters [49–52]. Another factor that can affect the association pattern, also related with the origin of populations, is the karyotypic number. While in European populations the karyotype typically consists on 29 (diploid) chromosomes, in North America populations this is usually 28 [53]. Here, Spanish samples presented 29 chromosomes, whereas Tasmanian samples presented 27. Different population origin and karyotype may also explain the signal found in Ssa21 in the RHA. According to this result, Ssa21 may play a noticeably role in sex determination in Spanish wild populations, which is compatible with the existence of an alternative population lineage in these populations. Although incorrect anchoring of markers to chromosomes is not disposable, if that was the case, this signal should also have appeared in the work by Kijas et al. [17]. In addition, residual tetraploidy resulting from the salmonid specific WGD may have influenced the evolution of their genomes leading to genomic instability [18]. An unstable state involves massive reorganizations of the chromosomes, including inversions, duplications and deletions, and may result in different sex chromosomes in different lineages [13, 14, 16, 18, 54].

Our findings also revealed sex-related genes in our candidate regions that had been previously identified in other species of the class Actinopterygii, including important maleness related genes such as *SDY* and genes from the *SOX* family. The sexually dimorphic on the Y chromosome is the master sex-determining gene in rainbow trout (*Oncorhynchus mykiss*) [8], and a male-specific Y-chromosome gene in the majority of salmonids [9]. Previous studies have determined that the expression of *SDY* is specific to males, being restricted to epithelial cells of the dorsal side of the testis and to some somatic cells adjacent to the germ cells [9, 16]. Indeed, overexpression of *SDY* in transgenic females of rainbow trout induces testicular differentiation [9]. Although the role of *SDY* in salmonids sex determination is still unclear, some authors have proposed that it is involved in the up-regulation of inhibitors of *CYP19A* (cytochrome P450 aromatase), preventing the accumulation of estrogens required for female development [30], in a similar way than *SRY* (sex-determining region Y) acts in mammals. Although it is not expected that the reference genome assembly carries the male-sex determining region of the *SDY* gene, since it is female-derived (assembly ICSASG_v2, [25]) the signals identified could be part of the male-female common region, described in Kijas et al. [17] or correspond to partial duplications, given the highly repetitive elements present in the Atlantic salmon genome. Using a comparative genomic approach, Voldoire et al. [31] demonstrated that the expansion of the *SOX* family after the teleost-specific WGD resulted in a high retention rate of paralogs, which followed lineage-specific evolutionary trajectories in teleost genomes. This is in agreement with the finding of several *SOX* genes in our regions.

In contrast, *R-spondin* genes present a conserved profile of enhanced expression in female vertebrates, and they are involved in ovarian determination and differentiation through the *WNT/β-catenin* signaling pathway [33]. This pathway is inhibited by the emerin gene, regulated by the *LMO7* gene [35, 36]. A recent study in medaka [34] demonstrated increased expression of genes of the *RSPO* family in the female gonad, suggesting a similar role in fish. Expression analysis in Nile tilapia, revealed that estrogen receptors mediate the development of undifferentiated XX gonads thorough estradiol activation [37] and in zebrafish, mutants with *ESR1* disrupted exhibited all-male phenotypes [38]. Also involved in the female reproductive process, *GNRH-R* activates the synthesis and secretion of gonadotropins in vertebrates through neuroendocrine control [40]. A previous study in seabream suggested that this gene has a role in meiosis-stimulating factor in the oocyte [41]. Finally, *FIGLA* is highly expressed in primary oocytes and has been localized to the ooplasm in medaka fish and coho salmon [43].

Today, it is generally accepted that most salmonids share the same master sex-determining gene, which has been moved through the action of transposable elements into different ancestral autosomes during the evolution of salmonids, resulting in alterative Y sex chromosomes [9].

Indeed, the first step in the evolution of a Y chromosome is the acquisition of a sex-determining locus on one of proto-sex chromosomes and, after that, the suppression of recombination between X and Y may favor the consolidation of the position of this gene. The role of recombination suppression between the X and Y chromosomes to resolve sexual conflict has been recently addressed by Wright et al. [55]. The authors analysed whole genome and transcriptome data in the guppy, a model for sexual selection with many Y-linked colour traits. Their results were consistent with a step-wise pattern of sex chromosome formation, suggesting that different regions of chromosome divergence can form independently within species. However, it is still debated why the sex chromosomes stopped recombining and how this process spread out over most part of the chromosomes. A recent in silico study by Mackiewicz et al. [56] revealed an association between the suppression of recombination and chromosome Y degeneration with the reproductive tactic, suggesting an enhancement of this effect in polygamous populations.

High-density SNP arrays are powerful tools to dissect QTLs and can highlight functional mechanisms underlying traits. However, appropriate analysis of dense marker information requires experimental designs with large sample sizes. In this sense, an important concern of our study was the reduced sample size available. Notwithstanding, our design allowed us to obtain significant estimates of whole-genome and chromosomal heritability, that remained significant even after applying strict Bonferroni correction. Thus, although our GWAS results may be interpreted with some caution, and validation of candidate SNPs in other genetic backgrounds is needed, the combination of both approaches (i.e. RHA and GWAS) provide valuable information for understanding the genetic basis of sex determination in Atlantic salmon.

## Conclusions

In summary, our results are compatible with previous studies that suggest a SD mechanism operating in Atlantic salmon where the *SDY* gene is the master sex determining gene, being Ssa02 the chromosome candidate for sex in this species. Interestingly, our findings regarding Ssa21 point towards an alternative population lineage in Spanish wild salmon, which inhabits the South distribution limit of the species in Europe. This study contributes to improve our understanding of an important trait in Atlantic salmon such as sex determination that has important implications both in terms of ecology and aquaculture production, and highlights the utility of the development and application of genomic tools in fish.

## Methods

### Samples and genotyping

A total of 203 sexually mature wild adult salmon from recreational fishery or recorded in trapping facilities (94 males and 109 females) were sampled between 2008 and 2013 from six Spanish rivers (Miño, Ulla, Eo, Sella, Urumea, Bidasoa) covering the distribution range of the species in Spain (from 41° 51′ 55.08″ N, 8° 52′ 10.99″ W to 43° 22′ 22″ N, 1° 47′ 31″ W). Since 1950, regulations have required that scale samples are collected from all salmon caught in the recreational fishery to determine fish age and growth and tissue samples (adipose fins) for DNA profiling analysis. In addition, routine sampling in trapping facilities allows for continuous monitoring and sampling of all sea returns and involves recording the length, weight and sex and taking scale samples for aging and a small portion of adipose fin with anesthesia for DNA profiling analysis.

Genomic DNA was purified from ethanol preserved adipose fins using an NZY Tissue gDNA Isolation kit (NZYtech), and quantity and purity assessed with a Nanodrop-1000 spectrophotometer. DNA samples were adjusted to a final concentration of 100 ng/μL and frozen until use. Morphological sex was confirmed by the successful amplification of the *SDY* intron gene (~ 200 bp) in all males and absence in all females using the primers *SDY* E1S1 and *SDY* E2AS4 [9, 57]. Samples were genotyped using an Affymetrix 220 K SNP array (ThermoScientific) for Atlantic salmon [27] according to manufactures recommendations. Genotypes from samples showing a dish quality control (DQC) < 0.82 or call rate < 0.97 were discarded. Only those data from SNPs classified as *Poly High Resolution*, with a call rate > 0.97 were used in our analysis. Unmapped SNPs and those with a minor allele frequency (MAF) < 0.01 were also removed. After applying these filters, data from 164,883 SNPs and 199 individuals (92 males and 107 females) remained available for analysis.

### Estimation of heritability

#### Genomic heritability analysis

Genomic heritability was estimated assuming a linear model of the form:

1 $$ \mathbf{y}=\boldsymbol{\upmu} +\mathbf{Zu}+\mathbf{e} $$

where **y** is the vector of phenotypic records (male, female), **μ** is the population mean of the trait **u** and **e** are vectors of random animal genetic and residual effects respectively, and **Z** is a design matrix allocating phenotypes to animals. Animal genetic effects were assumed to be distributed as *N* (0, **G** ${\sigma}_u^2$) where **G** is the genomic relationship matrix (GRM) for all fish and ${\sigma}_u^2$ is the additive genetic variance.

#### Regional heritability analysis

Regional heritability analysis was implemented to assess the contribution of different regions (in this case chromosomes) to the total additive genetic variance following Nagamine et al. [29]. For that, the random additive genetic effects were divided in two components: regional (chromosomal) genomic and residual whole-genomic additive genetic effects. For estimating the whole-genome component, all SNPs were used to build the GRM. For estimating the chromosomal component, 29 GRMs were built for each independent chromosome. The same whole-genome GRM was used for all analyses as in Nagamine et al. [29]. To test for significant chromosomal variance, likelihood ratio tests were performed by comparing the full model (including chromosomal and whole-genome additive genetic effects), with the reduced model (including only the whole-genome additive genetic effect, as in Eq. (1)). The full model was then of the form:

$$ \mathbf{y}=\boldsymbol{\upmu} +\mathbf{Zu}+\mathbf{Zc}+\mathbf{e} $$

where **y** is the vector of phenotypic records (male, female), **μ** is the vector of the population mean of the trait, **u** and **c** are vectors of whole-genome and chromosomal additive genetic effects, respectively, **e** is the vector of random residual effects, and **Z** is a design matrix allocating phenotypes to animals. Again, animal genetic effects were assumed to be distributed as *N* (0, **G** ${\sigma}_u^2$) where **G** is the genomic relationship matrix for all fish and ${\sigma}_u^2$ (${\sigma}_c^2$) is the additive genetic variance (computed from the whole-genome or from each chromosome, respectively).

### Genome-wide association studies

In order to avoid over-weighting the contribution of groups of correlated SNPs in the GWAS, we applied an additional filter based on linkage disequilibrium pruning. For that, we used a threshold value of 0.26, as this is the value (measured as the squared correlation coefficient, *r*^*2*^) at which linkage disequilibrium decreased half in the populations analysed (Additional file 4). Linkage disequilibrium pruning was performed using the software PLINK v1.9 [58] with the instruction *--indep-pairwise* using 0.26 as threshold, resulting in 36,625 SNPs.

GWAS was performed by regressing the standardised SNP genotypes on the trait using the following mixed model:

$$ \mathbf{y}=\boldsymbol{\upmu} +\mathbf{Xb}+\mathbf{Zu}+\mathbf{e} $$

which is basically the same model than in Eq. (1) but including a vector of fixed effects **b** that contains the substitution effect of the SNP, and where **X** is the design matrix for the fixed effect. The inclusion of the GRM in the model prevented potential bias for population structure [59]. A false discovery rate (FDR) multitest correction threshold at 5% level was employed to identify significant associations with the software Myriads [60].

Genetic values for RHA and GWAS were in all cases estimated using a relationship matrix as:

$$ {a}_{ij}=\frac{1}{M}\sum \limits_{k=1}^M{z}_{ik}{z}_{jk}, $$

where *a*_*ij*_ is the genetic relationship between individuals *i* and *j, M* is the number of markers and *z*_*ij*_ is the standardised genotype of individual *i* at marker *k*, defined as *z*_*ij*_ = (*s*_*ij*_ – μ_j_**)/** *σ*_*j*_, with *s*_*ij*_ being the number of reference alleles at locus *j* of individual *i*, *μ*_*j*_ **=** 2*p*_*j*_ and ${\sigma}_j=\sqrt{2{p}_j\ \left(1-{p}_j\right)}$ . *μ*_*j*_ and *σ*_*j*_ are the mean and the standard deviation of the reference allele at marker *j* among the individuals genotyped, defined as a function of the frequency of the reference allele (*p*_*j*_).

All models were solved assuming a dicotomic trait distribution (male / female) using restricted maximum likelihood (REML) with the software Dissect [61].

### Identification of candidate sex-related genes

The gene content within the ±0.5 Mb flanking regions from significant SNPs identified in the GWAS was interrogated to identify and characterise potential causative genes and variants using two approaches. We chose this distance because linkage disequilibrium at 0.5 Mb was still high in these populations (almost half its maximum value, Additional file 4). In a first step, we identified the genes in the Atlantic salmon genome annotation [25] that were located within candidate regions identified in this study. Secondly, we explored whether sex-associated genes previously described in *Salmo salar* [30] and other species of the Class Actinopterygii were located within (< 0.5 Mb from the candidate SNP) or in the vicinity (< 2.5 Mb, according to the extension of linkage disequilibrium, Additional file 4) of our candidate regions. The identification of sex-related genes in Actinopterygii was performed using the advanced search tool in NCBI database. Specific sex-determining pathway genes in Atlantic salmon were explored in the basis of the expression study by Lubieniecki et al. [30]. For the second approach, a total of 1050 genes were identified in 70 species. We restricted the search to 74 sex-related genes after removing duplicated gene names and genes in non-nuclear locations. In order to map the location of these 74 genes in the Atlantic salmon reference genome (assembly ICSASG_v2, [25]) we used the NCBI BLAST tool (blastn). Default search options were changed to discontiguous megablast, match/mismatch scores of 1,―1 and minimum gap cost (0 existence and 2 extension). Gene locations were inspected to overlap with candidate regions previously identified in the GWAS. Results were ordered by query cover and only alignments with a query cover > 50% or in chromosomes showing significant heritability for sex detected previously in RHA were considered.

## Supplementary information

**Additional file 1.** Information relative to SNPs significantly associated with sex identified in the GWAS

**Additional file 2. **Information relative to candidate regions identified in the GWAS for chromosomes Ssa02 and Ssa21contained in the *Salmo salar* annotation (Salmo_salar-annotation.gff3)

**Additional file 3. **Results of the BLAST analysis of sex-related genes previously identified in other species of the class Actinopterygii against the *Salmo salar* reference genome (assembly ICSASG_v2), indicating the corresponding chromosome match and position in *Salmo salar* and BLAST parameters

**Additional file 4. **Plot representing the decrease of linkage disequilibrium (measured as the squared correlation between alleles at different loci, *r*^*2*^) across physical distance in the chromosome

**Additional file 5.** Genotypic and phenotypic information used for the GWAS. Genotypes for the 36,625 (pruned) SNPs are coded as allele dosage (0, 1, 2) for the minor allele (A1) for each individual (first row). Phenotypic information for sex (1: male, 2: female) for each individual is also included (second row). Missing values are coded as NA

**Additional file 6.** Map information for the 36,625 (pruned) SNPs used for the GWAS, containing the corresponding chromosome, SNP name, physical position (in bp) and alleles (A1: minor allele, A2: major allele)

## Data Availability

The datasets analysed during the current study can be found in Additional file 5 and Additional file 6 of Supplementary Material.

## Abbreviations

BLAST: basic local alignment search tool
CYP19A: cytochrome P450 aromatase
DND: dead-end
DQC: dish quality control
ESR1: estrogen receptor 1
FDR: false discovery rate
FIGLA: folliculogenesis specific basic helix-loop-helix
GNRH-R: gonadotropin-releasing hormone receptor
GRM: genomic relationship matrix
GWAS: genome-wide association studies
LMO7: LIM domain only 7
MAF: minor allele frequency
QTL: quantitative trait loci
REML: restricted maximum likelihood
RHA: regional heritability analysis
RSPO1: R-spondin 1
SD: sex determination
SDY: sexually dimorphic on the Y-chromosome
SNP: single nucleotide polymorphism
SOX: SRY-type high mobility group box
SRY: sex-determining region Y
U2AF2A: U2 small nuclear RNA auxiliary factor 2a
WGD: whole genome duplication
WNT: wingless-related MMTV integration site 4
