# Supplementary figures and images for: Identification of genomic regions regulating sex determination in Atlantic salmon using high density SNP data
Source: BMC Genomics. 2019 Oct 22;20:764. doi: 10.1186/s12864-019-6104-4 (PMC6805462; doi:10.1186/s12864-019-6104-4)

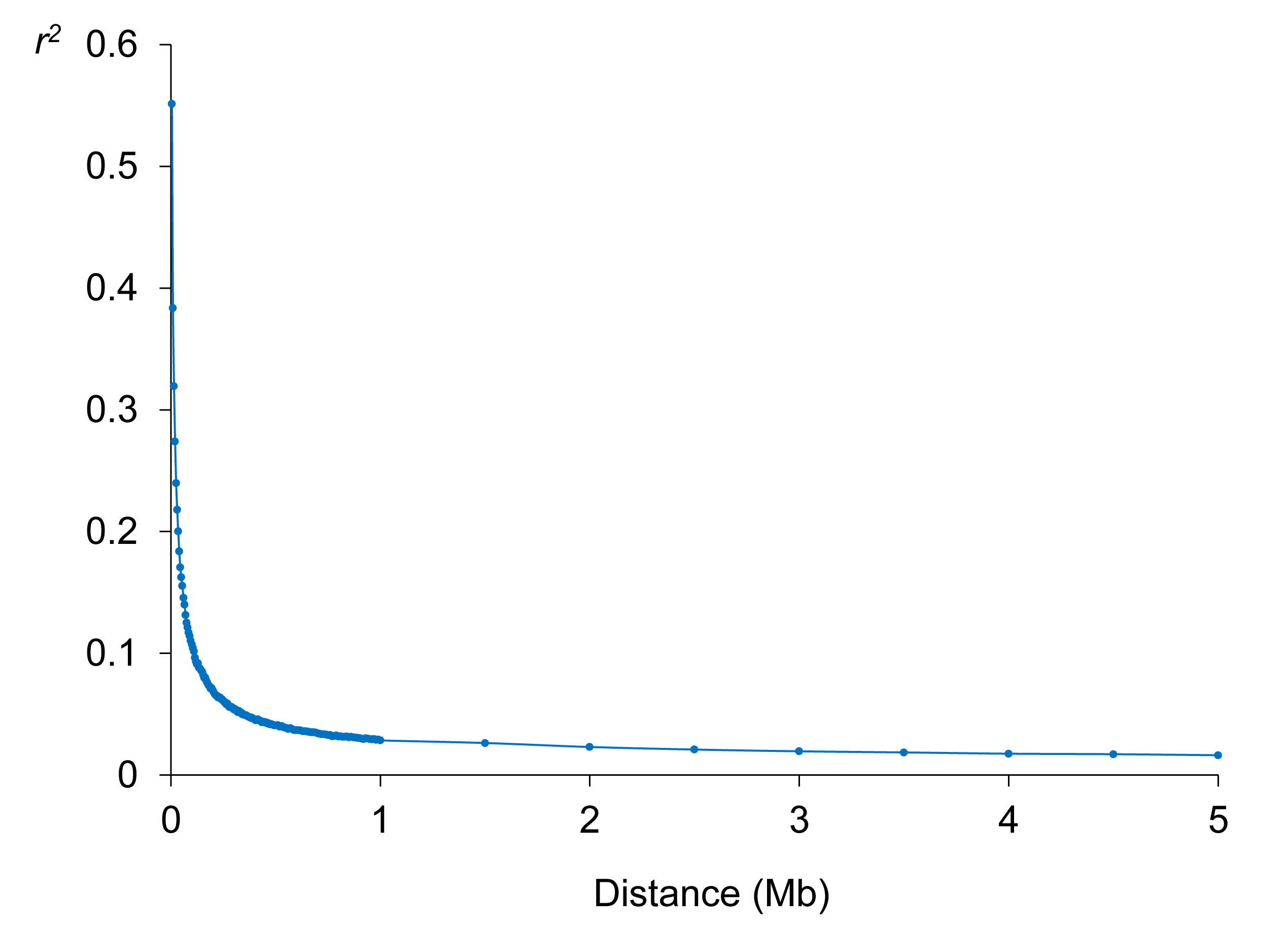

Supplement: Supplementary file 4 — Additional file 4. Plot representing the decrease of linkage disequilibrium (measured as the squared correlation between alleles at different loci, r2) across physical distance in the chromosome [file 12864_2019_6104_MOESM4_ESM.tif]
